# Membrane-bound *Gaussia* luciferase as a tool to track shedding of membrane proteins from the surface of extracellular vesicles

**DOI:** 10.1038/s41598-019-53554-y

**Published:** 2019-11-22

**Authors:** Mikołaj Piotr Zaborowski, Pike See Cheah, Xuan Zhang, Isabella Bushko, Kyungheon Lee, Alessandro Sammarco, Valentina Zappulli, Sybren Lein Nikola Maas, Ryan M. Allen, Purva Rumde, Bence György, Massimo Aufiero, Markus W. Schweiger, Charles Pin- Kuang Lai, Ralph Weissleder, Hakho Lee, Kasey C. Vickers, Bakhos A. Tannous, Xandra O. Breakefield

**Affiliations:** 1 Department of Neurology, Massachusetts General Hospital, Charlestown, MA 02129 USA; 2 Program in Neuroscience, Harvard Medical School, Boston, MA 02115 USA; 3 Department of Gynecology, Obstetrics and Gynecologic Oncology, Division of Gynecologic Oncology, Poznan University of Medical Sciences, 60-535 Poznań, Poland; 4 Department of Human Anatomy, Faculty of Medicine and Health Sciences, Universiti Putra Malaysia, 43400 Serdang, Selangor Malaysia; 5 Center for Systems Biology, Massachusetts General Hospital, Boston, MA 02114 USA; 6 Department of Comparative Biomedicine and Food Science, University of Padua, Padua, Italy; 7 Department of Neurosurgery, UMC Utrecht Brain Center, University Medical Center, Utrecht University, 3584 CX Utrecht, The Netherlands; 8 Department of Medicine, Vanderbilt University Medical Center, Nashville, TN 37232 USA; 9 Institute of Molecular and Clinical Ophthalmology Basel, 4031 Basel, Switzerland; 10 Present Address: Institute of Atomic and Molecular Sciences, Academia Sinica, Taipei, 10617 Taiwan; 11 Department of Radiology, Massachusetts General Hospital, Boston, MA 02114 USA; 12 Department of Systems Biology, Harvard Medical School, Boston, MA 02115 USA

**Keywords:** Secretion, Chromatography

## Abstract

Extracellular vesicles (EVs) released by cells play a role in intercellular communication. Reporter and targeting proteins can be modified and exposed on the surface of EVs to investigate their half-life and biodistribution. A characterization of membrane-bound *Gaussia* luciferase (mbGluc) revealed that its signal was detected also in a form smaller than common EVs (<70 nm). We demonstrated that mbGluc initially exposed on the surface of EVs, likely undergoes proteolytic cleavage and processed fragments of the protein are released into the extracellular space in active form. Based on this observation, we developed a new assay to quantitatively track shedding of membrane proteins from the surface of EVs. We used this assay to show that ectodomain shedding in EVs is continuous and is mediated by specific proteases, e.g. metalloproteinases. Here, we present a novel tool to study membrane protein cleavage and release using both *in vitro* and *in vivo* models.

## Introduction

Extracellular vesicles (EVs) are membrane-encapsulated vesicles released by cells into the extracellular space^1^. Cellular communication via EVs has been shown to contribute to many biological processes, including cancer cell proliferation and invasiveness^2–4^. In order to better understand the mechanisms of EV activity, researchers have developed molecular markers that should allow efficient tracking of EVs in culture and *in vivo*. We previously demonstrated that a membrane-bound version of *Gaussia* luciferase (mbGluc), also known as GlucB in previous reports, can be incorporated into the membranes of cells and EVs^5,6^. In these studies, EVs isolated by high-speed ultracentrifugation (UC) from cultured cells labeled with mbGluc were intravenously (i.v.) - injected into mice to monitor their biodistribution^6^. It is likely, however, that some methods of EV isolation, e.g. UC can distort EV structure, compromise EV integrity, and induce artificial protein and/or lipid aggregation and fusion events^7^. Additionally, the quantity of isolated EVs administered to mice is arbitrary and is often assessed as supraphysiological levels. To address these limitations, we initially planned to use cancer cells stably - transduced with mbGluc in a xenograft model in order to trace the fate of tumor-derived EVs in other tissues. In another study we used mbGluc to evaluate efficiency of immunocapture of tumor-derived EVs^8^, and we observed that the bioluminescence measured directly in serum was higher than that in EVs isolated by immunocapture. Although this could have resulted from inefficiency of EV immunocapture, the magnitude of the discrepancy suggested that the signal was present in other sources than just EVs. To determine if mbGluc is bound to non-EV material in the extracellular space and biofluids, we first evaluated mbGluc activity in conditioned medium collected from cancer cells without any EV isolation. Conditioned media was fractionated by sucrose/iodixanol gradients and mbGluc abundance was detected in fractions corresponding to EVs, but also was readily detected in the very low-density fractions, which are attributed to free proteins. These findings suggest that mbGluc has the potential to be secreted from cells in association with EVs or be cleaved from EVs as free protein of lipid-particles released from cells. These observations also suggest that this reporter system has the potential to monitor release of proteins from EV membranes, a unique phenomenon which has not been extensively investigated, likely due to the lack of tools to quantify such release. We used this knowledge to develop a novel assay to quantitatively track shedding of membrane proteins from the surface EVs both in culture and *in vivo*.

## Results

### Membrane-bound *Gaussia* luciferase is present on EVs

**Figure 1:**
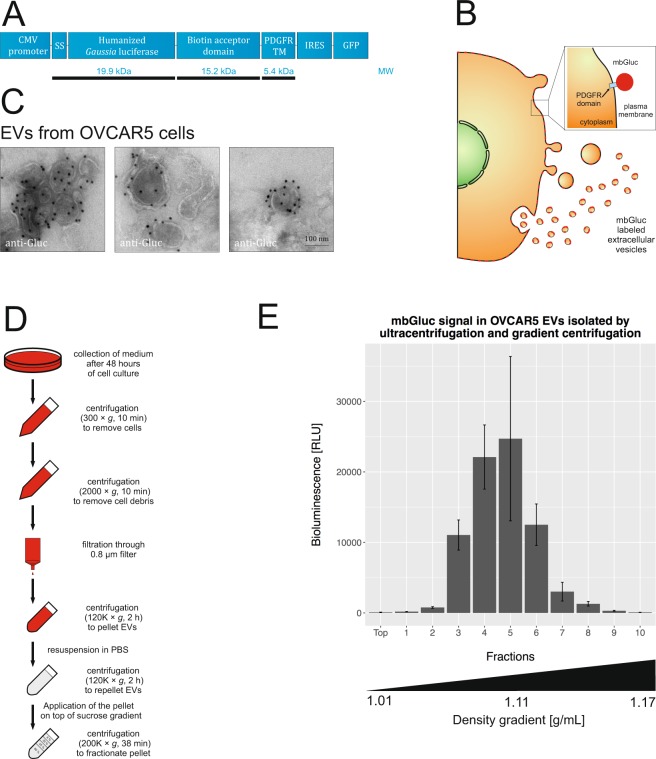
Membrane-bound *Gaussia* Luciferase Is Present on EVs. (**A**) *Gaussia* luciferase was fused to a transmembrane domain to present it on the plasma membrane and surface of EVs. SS – signal sequence, PDGFR TM - transmembrane domain of the platelet-derived growth factor receptor, IRES - internal ribosome entry site, GFP - green fluorescent protein, MW – predicted molecular weight of fragments of the fusion protein. (**B**) Schematic diagram to illustrate mbGluc labeling of cell membrane and EVs (based on the schematic presented previously^8^ by our group in Fig. 2A). (**C**) Transmission electron micrographs. EVs were isolated by UC from conditioned medium of OVCAR5 cells and immunolabeled with anti-Gluc antibodies; scale bar 100 nm. (**D**) Experimental flowchart of procedure to isolate EVs by UC and density gradient fractionation. (**E**) mbGluc activity in EVs isolated by UC and fractionated in sucrose density gradient (procedure in **D**); (representative of 2 experiments, bioluminescence in each fraction measured in 3 technical replicates).

The transmembrane domain of platelet-derived growth factor receptor was fused in-frame to the C-terminus of *Gaussia* luciferase (Gluc) to expose it on the cell surface, as reported (Fig. 1A)^5,6^. Consequently, extracellular vesicles (EVs) released from cells are labeled with mbGluc, also referred to as GlucB (Fig. 1B)^6^. Here we stably transduced a human ovarian cancer cell line, OVCAR5 with a lentivirus vector carrying a cDNA for mbGluc under the CMV promoter. Transmission electron microscopy and immunolabeling was used to visualize mbGluc on EVs isolated from conditioned medium by differential UC (Fig. 1C). To further corroborate the presence of mbGluc on EVs, we collected EVs by UC, washed them with PBS, prior to a subsequent UC step and then fractionated them on a sucrose density step-gradient (Fig. 1D). The peak bioluminescence signal was found at the approximate density of 1.11 g/mL, which is characteristic of EV fractions based on previous studies^9,10^ (Fig. 1E).

### mbGluc is abundant in low density fractions of sucrose step-gradient

**Figure 2:**
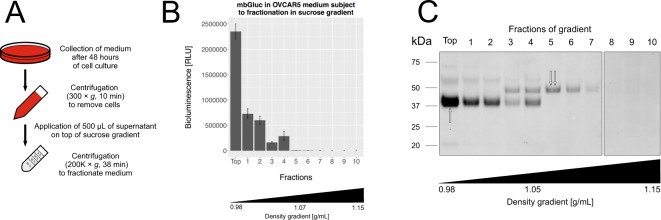
mbGluc Is Abundant in Low Density Fractions of Sucrose Step-Gradient. (**A**) Processing of a conditioned medium for density gradient fractionation without EV isolation. (**B**) mbGluc activity after fractionation (procedure in (**A**) of OVCAR5 conditioned medium in sucrose density gradient (representative of 3 experiments, each fraction measured in 5 technical replicates). (**C**) Western blot analysis of mbGluc protein following fractionation of OVCAR5 conditioned medium (representative of 3 experiments). The white bar divides lanes from separate membranes with samples from the same experiment processed according to the same procedure. The double arrow indicates 50 kDa band previously described as attributable to EV-bound mbGluc (compare to Fig. 3C). Single arrow points to a band of approximately 40 kDa. Full picture of the gel is presented in Fig. S5A.

**Figure 3:**
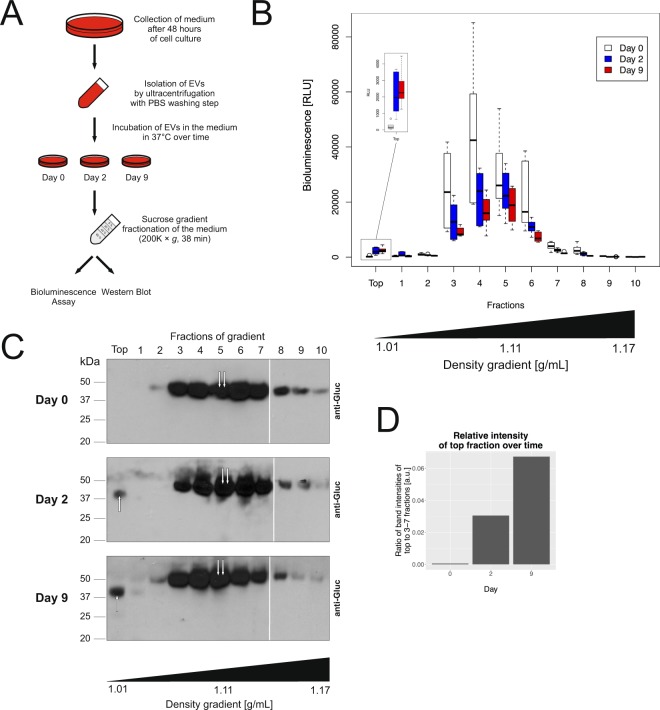
mbGluc as a Measure of Release of EV-bound Membrane Proteins. (**A**) An experimental flowchart of procedure to isolate EVs by UC and incubate them for 0, 2 and 9 days in medium supplemented with no FBS at 37 °C in a 5% CO_2_ incubator. (**B**) mbGluc activity measured after fractionation of EVs derived from OVCAR5 mbGluc-positive cells in sucrose density step-gradient following incubation at 37 °C for 0, 2 and 9 days. The insert represents measurements in the top fraction with a modified scale on Y-axis. (**C**) Western blot analysis of mbGluc protein in density gradient over time. The white bar divides lanes from separate membranes with samples from the same experiment processed according to the same procedure. The size of mbGluc in the low-density “Top” fraction was smaller (approximately 40 kDa, single arrow) in comparison to mbGluc found in EV fractions (fraction 3–6; approximately 50 kDa, double arrow). Full pictures of the gels are presented in Fig. S5B–D. (**D**) Intensity of “Top” fraction band calculated relative to intensity of fractions 3–7.

In order to evaluate whether mbGluc could be used as a reporter in a xenograft model to track tumor EV distribution *in vivo*, we tested its release profile from cells. Conditioned medium from cells was directly applied on top of a sucrose step-gradient followed by centrifugation with fractions collected from the top down (Fig. 2A). Surprisingly, most of the luciferase activity was found in the top fraction, which had the lowest density (Fig. 2B). Western blot analysis confirmed in the middle fractions (3–6) the presence of a 50 kDa band previously described as attributable to EV-bound mbGluc^6,8^ (double arrow, Fig. 2C, compare to Fig. 3C). We observed, however, a more intense band of approximately 40 kDa in the top fraction (single arrow, Fig. 2C). Its lower molecular weight suggested that the exposed part of the mbGluc protein was released from the surface of the EVs with a fragment remaining in the EV membrane. This profile of the mbGluc signal in sucrose fractions was also found in conditioned medium from other cell lines (Fig. S1A,B). When the same experiment was performed in the medium without fetal bovine serum (FBS), the total mbGluc bioluminescence signal was lower; however, the distribution of the signal in the gradient fractions was similar to FBS-containing medium (Fig. S1C,D). This implied that constituents of FBS enhanced a process leading to transfer of mbGluc activity to structures detected in the top fraction. It is possible that in the medium without FBS more mbGluc remained on the cell surface without being released to the extracellular space. This impact of FBS was further investigated in the experiments described below.

Alternatively, we also considered the possibility that EVs not concentrated by UC may distribute in a different way across the sucrose step-gradient. For example, they might require a longer centrifugation time to reach equilibrium. Indeed, an extension of centrifugation time from 38 minutes to 18 hours resulted in a shift of the signal from the top fraction to fraction 3 (Fig. S1E). An alternative method to isolate EVs by gradient fractionation involves loading the sample at the bottom of the tube after mixing with the solution with the highest sucrose concentration. In that case, we observed that the mbGluc signal did not float to the top-fraction but remained in fraction 6 (corresponding to the top of mixed 60% sucrose and sample solution) (Fig. S1F, gradient before centrifugation – Fig. S1G). Some studies suggest that sucrose solutions have less accurate density resolution for EV isolation than iodixanol^11^. We observed that mixing conditioned medium with 60% iodixanol at the bottom and loading of 40% iodixanol solution at the top resulted in the movement of the mbGluc activity to a less dense fraction 4 (Fig. S1H). The mbGluc signal did not float up from that fraction after 18 hours of centrifugation (Fig. S1I). Although EVs which were not ultracentrifuged could have distinct biophysical properties from those that were, these observations raised the possibility that a substantial portion of mbGluc might be present in particles smaller than typical EVs, i.e. <70 nm^12,13^.

### mbGluc as a measure of release of EV-bound membrane proteins

We hypothesized that low-density mbGluc might reflect release of the active portion of this protein from the surface of EVs into the extracellular space. To evaluate this possibility, we isolated EVs by UC with an additional washing step to remove any remnants of signal represented in the low density fraction and incubated these EVs in medium supplemented with no FBS at 37 °C for 0, 2 or 9 days in a 5% CO_2_ incubator (Fig. 3A). The medium with EVs spiked-in was subsequently fractionated on a sucrose step-gradient. We observed that the bioluminescence signal in the fractions corresponding to EVs (fractions 3–6) decreased in the course of time, probably reflecting protein release and degradation (Fig. 3B). Simultaneously, the activity of mbGluc in the top fraction increased over time (Fig. 3B). Western blot analysis of fractions confirmed an increasing amount of the mbGluc protein in the top fraction over time (single arrow, Fig. 3C,D). Interestingly, the size of this low-density form of mbGluc was smaller (approximately 40 kDa, single arrow, Fig. 3C) in comparison to mbGluc found in EV fractions (fraction 3–6; approximately 50 kDa, double arrow, Fig. 3C). The size of the protein that appeared over time (approximately 40 kDa) was similar to the most intense band observed in the top fraction of conditioned medium (Fig. 2C). This experiment demonstrated that some of the membrane protein originally found only on EVs (Fig. 3C, Day 0) in the course of time moved to the pool of the low-density fraction to structures that were no longer bound to EVs (Fig. 3C, Day 2 and 9). This transition to a low density Gluc was even more striking, when EVs isolated by UC were incubated in the medium supplemented with 5% EV-depleted FBS, suggesting that components in serum accelerate this process (Fig. S2). It is worth noting that the difference in size between those two forms of mbGluc protein is close to the size of its transmembrane domain (5.4 kDa). Therefore, this observation supports the hypothesis that the low-density fraction of mbGluc may reflect the release of part of the protein from the EV membranes, with the transmembrane fragment remaining in the EV membrane.

### mbGluc is mainly present in small particles (<70 nm) fractionated by size exclusion chromatography

**Figure 4:**
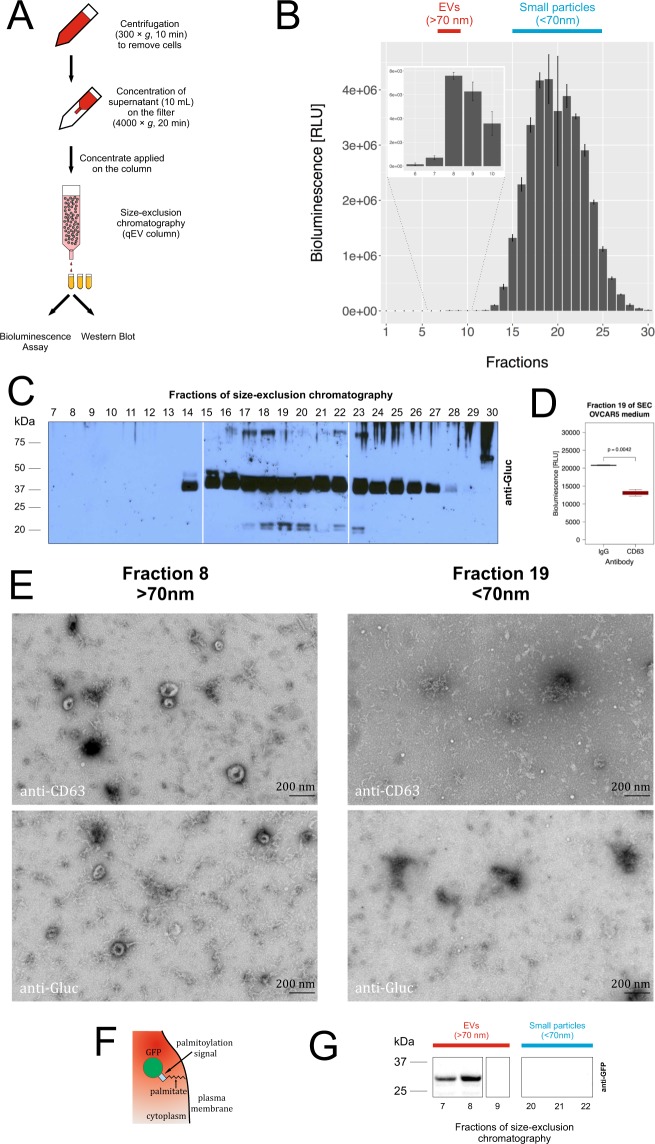
mbGluc Is Mainly Present in the Small Particle – Fractions by Size Exclusion Chromatography (SEC). (**A**) Schematic of procedure to fractionate conditioned medium by SEC. (**B**) mbGluc activity in SEC fractions (qEV column) after fractionation of OVCAR5 conditioned medium (Procedure in (**A**); (representative of 2 experiments, each fraction measured in 3 technical replicates). The insert represents fractions 6–10 with a modified scale on Y axis that ranges from 0 to 8,000 RLU. (**C**) Western blot analysis of mbGluc protein following fractionation of OVCAR5-mbGluc+ medium by SEC (representative of 2 experiments). The main mbGluc band was at 40 kDa, similarly to that observed in the top fraction after sucrose step-gradient fractionation of the conditioned medium (compare to Fig. 2C). Two other less intense bands are at 20 kDa (which is close to the approximate size of free non-membrane bound Gluc without the biotin acceptor domain – 19.9 kDa) and 80 kDa (twice the size of the dominant 40 kDa band). The white bar divides lanes from separate membranes with samples from the same experiment processed according to the same procedure. Full picture of the gel is presented in Fig. S5E. (**D**) mbGluc activity in structures captured by anti-CD63 or IgG (unspecific binding) antibody in fraction No 19 which is representative of small particles fractions (fractionation of OVCAR5 medium in qEV column). (**E**) Transmission electron microscopy (TEM) of fraction 8 and 19 of SEC (qEV column). Material from the fractions was directly applied on the grid and immunolabeled with either anti-CD63 or anti-Gluc antibodies, scale bar: 200 nm. (**F**) Schematic diagram of cell membrane and EV labeling with palmGFP. (**G**) Western blot analysis of palmGFP protein following fractionation of OVCAR5-palmGFP + medium by SEC in fractions representative of EVs (7–9) and particles smaller than 70 nm (20–22). The white bar divides lanes from separate membranes with samples from the same experiment processed according to the same procedure. Full picture of the gel is presented in Fig. S5F.

Since UC followed by sucrose-gradient fractionation may potentially distort the structure and biophysical properties of EVs, thereby disturbing the distribution of mbGluc, we used another method of EV isolation - size exclusion chromatography (SEC) using qEV columns (Fig. 4A). According to the manufacturer’s specifications, the column separates structures larger than 70 nm (fractions 7–9) from those smaller than 70 nm (approximate fractions 15–25). An analysis of mbGluc activity in OVCAR5 conditioned medium across SEC fractions revealed a predominant signal in the spectrum of structures smaller than 70 nm, and a smaller peak in the putative EV fractions (7–9) (Fig. 4B). This analysis corroborated that although some mbGluc was present on EVs in conditioned medium, most of the luciferase activity derived from it was found in smaller structures. The small particle fractions (<70 nm) contain free proteins, protein aggregates, lipoproteins, products of membrane degradation and potentially EVs smaller than 70 nm. An analysis of SEC fractions by western blot confirmed that the mbGluc protein is mainly present in the small particle fractions (Fig. 4C). The major Gluc band was at 40 kDa, similarly to that observed in the top fraction after sucrose step-gradient fractionation of the conditioned medium (Fig. 2C) and over time in the fractionation of EVs isolated by UC from the conditioned medium (Fig. 3C). The size of that band was close to that of mbGluc without the transmembrane domain, corresponding to Gluc (19.9 kDa) and the biotin acceptor domain (15.2 kDa). We also noticed two other less intense bands with sizes of approximately 20 kDa (which is close to the approximate size of free non-membrane bound Gluc without the biotin acceptor domain – 19.9 kDa) and 80 kDa (twice the size of the dominant 40 kDa band). This suggests that the released protein may be subject to dimerization. It is also possible that the small particle fractions contain EVs smaller than 70 nm in diameter. Since the tetraspanin CD63 is present on many EVs^11^, we explored immunocapture with anti-CD63 antibodies in the small particle fraction (fraction 19). However, this revealed no enrichment of mbGluc activity as compared to non-specific IgG binding. This indicated that there were no small membrane structures containing both CD63 and mbGluc (Fig. 4D). To visualize the structures in the different fractions of EVs and particles resolved by SEC, we performed transmission electron microscopy (TEM) using antibodies against Gluc and CD63. In fraction 8 (one of the fractions representing EVs) we observed the presence of EVs of approximately 100–150 nm in diameter (Fig. 4E, left panels). The immunolabeling confirmed the presence of both CD63 and mbGluc on EVs in that fraction. Fraction 19 (one of middle fractions representing particles smaller than 70 nm) contained numerous structures with diameters ranging from approximately 20–30 nm that were not labeled for either CD63 or mbGluc. These smaller particles most likely corresponded to low density lipoproteins based on their size (Fig. 4E, right panels). Dispersed gold particles were not associated with any identifiable structures in fraction 19.

Membrane proteins with one transmembrane and one extracellular domain, such as mbGluc may undergo cleavage of the exposed part. That would result in a freely floating Gluc and detection of the signal in the small particle fraction. To test if the orientation in the plasma membrane of a reporter protein determined its mode of release, we analyzed release of green fluorescent protein conjugated with a palmitoylation signal – palmGFP (Fig. 4F). This type of reporter protein modification induces binding of the palmitoylated protein on the internal leaflet of the plasma membrane and secretion with EVs^14^. One could expect that membrane proteins configured in this way would not be affected by external proteases and thus not subjected to release by cleavage. To test this hypothesis, we fractionated the OVCAR5-palmGFP conditioned medium by SEC. In this experiment, as detected by western blot analysis, we observed signal only in the EV fractions but not in the small particle fractions (Fig. 4G). That suggested that a protein had to be present on the external side of the plasma membrane to be susceptible to transfer to the small particle fraction. That observation supported the hypothesis that mbGluc transformation was due to proteolytic cleavage of its ectodomain by extracellular proteases.

### Level of expression does not affect mode of release of mbGluc

We also considered the possibility that the observed profile of mbGluc release resulted from a high level of expression in transduced cells. It is possible that due to limited capacity, cells are only able to incorporate a specified amount of artificially expressed protein into the plasma membrane. In that case, excessive protein that does not get incorporated in the membrane might be released into the extracellular space in a non-membrane-bound form. To test this hypothesis, we compared SEC profiles of mbGluc release from cells transduced with different titers of lentivirus expressing mbGluc. We observed that with a lower multiplicity of infection (MOI) of lentiviral transduction, the mbGluc signal dropped in all SEC fractions (Fig. S3A–C). At all multiplicities of infection (MOIs) we observed that the dominant signal was associated with the small particle fractions, with a smaller peak in the EV fraction (Fig. S3A-C). This indicates that the level of expression does not affect the mode of release of mbGluc, which is mainly found in small particle fractions regardless of level of expression.

### mbGluc is also present as a free protein, independent of lipid carriers

**Figure 5:**
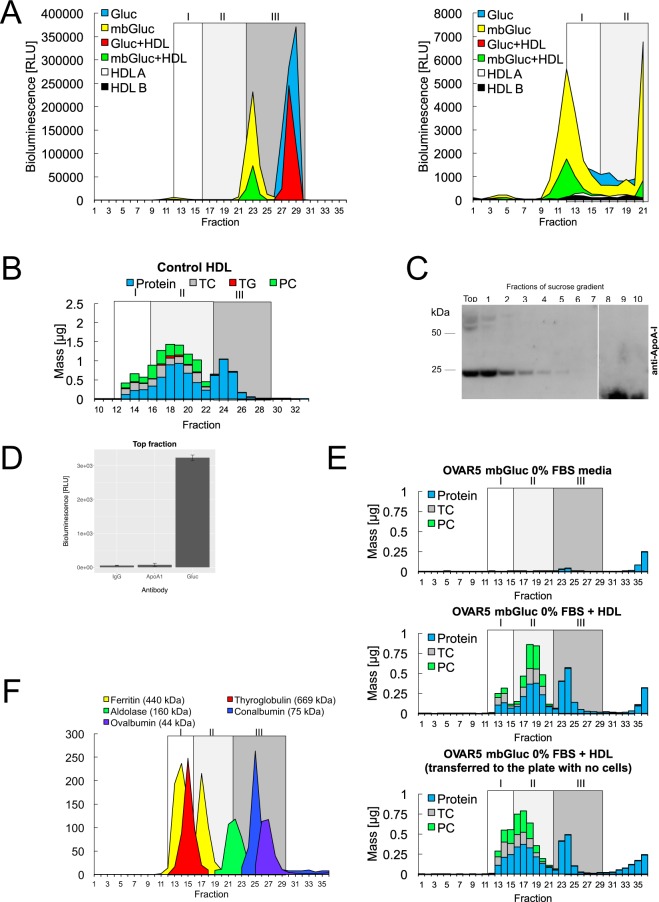
mbGluc Is also Present as a Free Protein, Independent of Lipid Carriers. (**A**) Fast protein liquid chromatography (FPLC) of conditioned medium derived from OVCAR5-mbGluc+, OVCAR5-Gluc+ (Gluc that is not fused to transmembrane domain and is secreted freely into extracellular space), non-conditioned medium with human HDL spiked-in (HDL-A and HDL-B are replicates of samples with HDL) as well as OVCAR5-mbGluc+ and OVCAR5-Gluc+ media mixed with HDL. Left panel – all fractions, right panel – fractions 1–21 with reduced scale on Y axis. (**B**) Composition of human HDL fractionated by FPLC, TC – total cholesterol, TG – triglycerides, PC – phosphatydylcholine (data representative of 2 experiments). (**C**) Western blot analysis of apolipoprotein A-I (ApoA-I) protein (marker of HDL) following fractionation of OVCAR5 conditioned medium. The white bar divides lanes from separate membranes with samples from the same experiment processed according to the same procedure. Full picture of the gel is presented in Fig. S5G. (**D**) mbGluc bioluminescent activity in structures captured by anti-ApoA-I, anti-Gluc or IgG (unspecific binding) antibody in top fraction of sucrose density-step gradient following fractionation of OVCAR5-mbGluc+ medium. (**E**) Composition of OVCAR5-mbGluc+ conditioned medium (upper panel), OVCAR5-mbGluc+ conditioned medium with addition of HDL (middle panel) and OVCAR5-mbGluc+ preconditioned medium mixed with HDL in the absence of cells (lower panel) in FPLC fractions. (**F**) FPLC fractionation of standard proteins to determine the fractionation and distribution of high-molecular weight standards. Zone I corresponds to particles of approximately 25–75 nm in diameter (e.g. VLDL/LDL/small EVs), Zone II corresponds to particles of approximately 7–12 nm in diameter (e.g. HDL) and Zone III corresponds to particles smaller than 7 nm in diameter.

The particles bearing mbGluc in conditioned medium were further analyzed using sequential SEC (triplicate in-line Superdex 200 increase resin columns (GE) with an Atka Pure (GE) fast protein liquid chromatography system (FPLC)) that enabled high resolution in the range of small particles. FPLC fractionation revealed that the major signal for mbGluc activity (fraction 23) is detected in fractions associated with small high-density lipoproteins (HDL) or potentially small non-HDL protein complexes (Fig. 5A, left panel). A comparison with the fractionation of human HDL under the same protocol demonstrated partial overlap of mbGluc bioluminescence (fraction 23) with HDL particles, potentially nascent pre-β HDL or lipid-poor HDL apolipoprotein A-I (ApoA-I) – ApoA-I is the main structure-function protein on HDL. For example, the mbGluc signal over-lapped with HDL fractions that showed reduced cholesterol and phospholipids (i.e. phosphatidylcholine) compared to earlier fractions associated with spherical HDL containing abundant cholesterol and phospholipids (Fig. 5B). Of note, we did detect a much smaller signal of mbGluc activity in fractions associated with the larger apolipoprotein B-containing lipoprotein classes (e.g. VLDL/LDL) and EVs (e.g. fraction 12) (Fig. 5A, right panel). That peak was absent when non-membrane bound Gluc was analyzed, confirming some EV labeling with mbGluc. Nevertheless, we posited that the partial overlap between the highest mbGluc activity and small HDL particles or free proteins (e.g. fraction 23) might reflect a transfer of mbGluc from EVs to HDL. Consistent with this hypothesis, ApoA-I, was most abundant in the top fraction of the sucrose step-gradient (Fig. 5C), similar to where the bulk of activity derived from mbGluc appears (Fig. 2B,C). To evaluate a possible interaction between mbGluc and HDL, we measured luciferase activity in FPLC fractions in medium with no FBS (as FBS contains bovine HDL) and compared it to a parallel sample with added human HDL; however, we failed to detect an increase in signal with the addition of the HDL (Fig. 5A). To further investigate this possibility, we isolated HDL from the top fraction of the sucrose step-gradient using anti-ApoA-I antibodies, but also failed to observe a significant enrichment of Gluc activity over nonspecific IgG binding; in contrast to positive controls with anti-Gluc antibody (Fig. 5D). These observations indicated that derivatives of mbGluc are not likely associated with HDL, but share certain physical and chemical properties with HDL, e.g. density and size. To further characterize the fraction containing mbGluc derivatives we determined the protein and lipid components in the FPLC fractions. The fraction most enriched for the mbGluc activity, fraction 23, was found to be protein-rich, cholesterol-poor, triglyceride-poor, and phosphatidylcholine-poor; characteristic of free protein in plasma (Fig. 5E). This finding suggests that the majority of mbGluc activity is not associated with bilayer phospholipid membranes of EVs or single phospholipid monolayers of HDL, but likely exists in the form of free protein and/or lipid-poor protein complexes. To characterize the size of those complexes we applied high molecular weight protein standards to separation by FPLC (Fig. 5F). The majority of mbGluc activity was detectable in the fraction corresponding approximately to the size between aldolase (160 kDa) and conalbumin (75 kDa) standards, suggesting that cleaved mbGluc proteins may form dimers and higher order oligomers, e.g. a 80 kDa dimer of 40 kDa protein fragments. Taken together, these results support that mbGluc, after being released is no longer bound to phospholipid bilayer (membrane) structures, but is cleaved into 40 kDa fragments with the potential to form protein complexes and dimers.

### mbGluc provides a quantitative measure of shedding of proteins from EV membranes *in vitro* and *in vivo*

**Figure 6:**
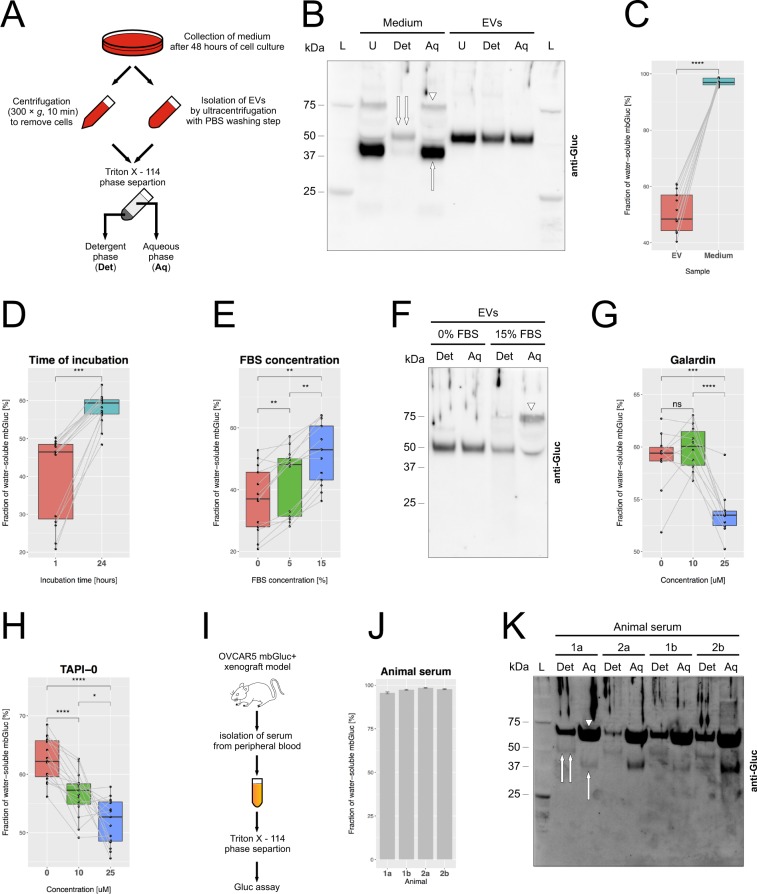
mbGluc Provides a Quantitative Measure of Shedding of Proteins from EV Membranes *in Vitro* and *in Vivo*. (**A**) Schematic of procedure to separate water-soluble freely floating (“Aq”) from detergent-soluble with transmembrane domain (“Det”) proteins by TritonX – 114 (TX-114) method. (**B**) Western blot of mbGluc protein isolated from OVCAR5-mbGluc+ conditioned medium and EVs (isolated by UC) subjected to TX-114 phase separation. The water-soluble form of mbGluc (aqueous phase) was present as proteins of approximately 40 kDa (single arrow) and 80 kDa (arrowhead). In the detergent phase, mbGluc band corresponded to approximately 50 kDa, the same as detected in unprocessed EVs (compare to Fig. 3C). L – ladder, U – unprocessed sample (no TX-114 separation), Det – detergent phase of TX-114 separation, Aq – aqueous phase of TX114 separation. (**C**) Fraction of water-soluble mbGluc based on bioluminescence in OVCAR5-mbGluc+ conditioned medium and EVs isolated by UC. EVs were fractionated by TX-114 procedure with two separation cycles. (**D**) Fraction of water-soluble mbGluc in the medium (with 0% FBS) with EVs incubated for 1 and 24 hours at 37 °C in a 5% CO_2_ incubator. (**E**) Fraction of water-soluble mbGluc in EVs incubated for 1 hour in the medium supplemented with 0, 5 and 15% FBS at 37 °C. (**F**) Western blot of mbGluc protein in medium with EVs incubated for 1 hour in the medium with 0 and 15% FBS subjected to TX-114 phase separation. FBS promoted the appearance of an 80 kDa protein, observed previously in the medium (arrowhead, lane “15% FBS, Aq”, compare to (**B**). (**G**) Fraction of water-soluble mbGluc in EVs incubated for 24 hours in medium supplemented with 5% FBS and a broad-spectrum MMPs inhibitor GM6001 (galardin). (H) Fraction of water-soluble mbGluc in EVs incubated for 24 hours in the medium supplemented with 5% FBS and TAPI-0, a potent inhibitor of MMPs and ADAM17. (**I**) Schematic of serum isolation from mice bearing OVCAR5-mbGluc+ tumors. Serum was subjected to TX-114 procedure and analyzed by Gluc assay. (**J**) Fraction of water-soluble mbGluc in serum from OVCAR5-mbGluc+ mice 1 and 2 (two separate serum samples “a” and “b” were analyzed from each animal, bioluminescent measurements were done in 3 technical replicates). (K) Western blot of mbGluc protein in serum from OVCAR5-mbGluc+ mice 1 and 2 (technical replicates a and b in each) separated by TX-114 method. The signal in aqueous phase was mainly coming from an 80 kDa protein (arrowhead) as well as from 40 kDa band (single arrow, lanes “Aq”). A simultaneous presence of 80 kDa and 40 kDa suggests that fragments of mbGluc may form dimers after being cleaved-off. The 50 kDa band characteristic of EV-bound mbGluc was detected in detergent phases from all serum samples (double arrow, lanes “Det”). For all experiments in B-H, EVs were isolated by two UC steps (120 K × *g*, 2 hours). The pellet was dissolved in RPMI medium and incubated at 37 °C under conditions specified above for 24 hours (unless specified otherwise). The medium was subsequently separated by TX-114 into water-soluble and detergent-soluble phase. Plots in C-H demonstrate data from 3 experiments, in each there were 5 technical replicates of Gluc bioluminescence.

Based on FPLC analysis, the mbGluc-derived signal is mainly present in protein complexes. It could be expected that freely floating proteins are water-soluble in contrast to the intact mbGluc which is imbedded in the membrane of EVs. To distinguish those two forms of mbGluc we fractionated the samples using Triton X–114^15^. This detergent is able to separate water-soluble (non-membrane-bound) proteins in the aqueous phase from integral membrane proteins in the lipid or detergent phase (*ibid*.). We applied Triton X-114 phase separation to both OVCAR5 conditioned medium (after removal of cells) and EVs isolated by UC with additional washing steps (Fig. 6A) followed by western blot analysis. The water-soluble form of mbGluc (aqueous phase) was present as proteins of approximately 40 and 80 kDa, similarly to the major signal detected in the small-particle fractions in SEC experiments (lane “Medium, Aq”, 40 kDa – single arrow, 80 kDa – arrowhead, Fig. 6B, compare to Fig. 4C). In contrast, immunoreactive protein found in the detergent phase corresponded to approximately 50 kDa, the same as detected in unprocessed EVs (lane “Medium, Det”, double arrow, Fig. 6B, compare to Fig. 3C). Surprisingly, we detected mbGluc protein in both detergent and aqueous phases after separation of EVs (lane “EVs, Det” and “EVs, Aq”, Fig. 6B). In principle, all membrane-bound proteins should be found only in the detergent fraction. We hypothesized that this discrepancy may have resulted from imperfect separation and we increased the number of centrifugation steps from two to four in the Triton X-114 procedure. This resulted in the detection of full length mbGluc protein only in the detergent fraction (Fig. S4).

We considered using the bioluminescence assay of mbGluc as a quantitative measure of protein transfer from the membrane-bound to the water-soluble form. Indeed, we observed that bioluminescence from water-soluble mbGluc was markedly higher in the medium (after removal of cells) than in EVs (Fig. 6C). We decided to use this assay to characterize the process of membrane protein shedding from the surface of EVs into the medium. The transfer to the water-soluble form was time dependent, since after 24 hours we observed more water-soluble Gluc activity than after 1 hour of incubation (Fig. 6D). We hypothesized that the process of protein shedding might be dependent on proteases that cleave off extracellular parts of membrane proteins. We demonstrated that mbGluc associated with EVs isolated by UC and kept in medium supplemented with increasing concentrations of FBS underwent more shedding of Gluc activity (Fig. 6E). This observation was consistent with our previous conclusions that more Gluc activity was detected in the top fraction of sucrose step-gradients when treated with higher concentration of FBS (Fig. S2). Given that FBS is rich in proteases, this observation supported the view that mbGluc was cleaved-off the membrane by proteolysis. Western blot analysis revealed that higher concentration of FBS promoted the appearance of an 80 kDa protein, observed previously in the medium (arrowhead, lane “15% FBS, Aq”, Fig. 6F, compare to Fig. 6B). We interpreted the higher size of the protein as an aggregation or dimerization of Gluc after cleavage. In order to analyze the mechanisms involved in membrane protein shedding, we applied two proteinase inhibitors to the medium. We found that a broad-spectrum matrix metalloproteinase (MMPs) inhibitor GM6001 (also known as galardin) reduced the cleavage of mbGluc (Fig. 6G). We also showed that Gluc shedding was suppressed in a dose-dependent manner by the TNF-α Protease Inhibitor-0 (TAPI-0) which is a potent inhibitor of MMPs and membrane-bound disintegrin and metalloproteinase 17 (ADAM17; Fig. 6H).

Finally, to investigate the process of protein cleavage *in vivo*, we established murine OVCAR5 mbGluc-positive xenograft model by intraperitoneal injection of cancer cells (Fig. 6I). After 4–5 weeks of tumor growth, we collected the serum and fractionated it into the detergent and aqueous phase using Triton X-114. We observed that 95–98% of the Gluc activity was present in the water-soluble form (Fig. 6J). Western blot analysis revealed that in all samples the intense signal in the aqueous phase was derived from an 80 kDa protein (arrowhead, Fig. 6K) with simultaneous presence of 40 kDa band (single arrow, Fig. 6K). The simultaneous presence of 80 kDa and 40 kDa bands suggests that fragments of mbGluc may form dimers after being cleaved-off. The 50 kDa band characteristic of EV-bound mbGluc was detected in detergent phases from all serum samples (double arrow, Fig. 6K). It is worth mentioning that the water-soluble form of Gluc found in the animal serum could be derived both from cells and EVs. Both findings in *in vitro* and *in vivo* models provide insight into mechanisms involved in mbGluc cleavage and demonstrate that the bioluminescence Gluc assay is suitable for studying factors affecting protein shedding.

## Discussion

An initial aim of this study was to analyze biodistribution of EVs released *in vivo* in the course of tumor growth. To address that question we used a highly sensitive reporter protein – mbGluc previously developed by our group^5,6^. We confirmed that EVs isolated by UC were labeled with mbGluc. While analyzing the entire spectrum of mbGluc bioluminescence, we noted, however, that in addition to activity attributable to EVs, there was also a significant signal coming from particles smaller than EVs. The goal of this work was to characterize the nature of this unexpected mbGluc activity. We concluded that mbGluc undergoes ectodomain shedding and thanks to the quantitative nature of bioluminescence assay, this reporter protein may be used to study cleavage of membrane proteins.

There are many subpopulations of EVs and their characterization is a subject of ongoing debate^11,16,17^. The size of EVs concentrated by 120,000 × *g* centrifugation (following a 2,000 x g centrifugation and discard of that pellet) ranges from approximately 50 to 120 nm^18,19^. Structures present in the extracellular space that are smaller than this range include, among others, lipoproteins, protein complexes, potentially smaller EVs and recently described exomeres^20^. We considered that mbGluc might be released in one of those forms or transferred from EVs to those structures. Indeed, we observed that HDL protein marker, ApoA-I, was detectable in similar fractions as mbGluc activity. We also detected lipoprotein-like structures in the SEC fraction, where the mbGluc signal was the highest. We observed, however no enrichment of Gluc signal upon immunocapture with anti-ApoA-I antibody specific to HDL. Analysis of a high-resolution FPLC also demonstrated that HDL (7–12 nm) were bigger than Gluc-bearing structures. Exomeres, that are around 35 nm in diameter, also fell into a distinct range of size^20^. An analysis of the contents of the mbGluc-rich FPLC fraction (fraction 23, Fig. 5B) indicated proteins as major constituents with hardly detectable presence of lipids. That observation spoke against the hypothesis that mbGluc was in smaller EVs since they would contain more lipids as membrane-associated structures. Our experiments performed by various methods (sucrose/iodixanol gradient fractionation, SEC, FPLC) suggested that the Gluc signal was coming from the fraction containing protein complexes. Proteins detected in the extracellular space contain, among others, RNA-binding proteins (RBPs), including miRNA-binding Argonaute Ago^21^ and dsDNA-binding histones^16^. It has been demonstrated that those protein complexes are almost exclusively present in the non-vesicular fraction^16^. It cannot be excluded that mbGluc is partly secreted as a primary protein complex. In this study, however, we observed that mbGluc, which had been initially bound to EV surface, transformed in the course of time into a protein complex (Figs 3, 6). A change in size of mbGluc protein implied that its transmembrane portion remained in the membrane. This process was stimulated by FBS in a dose-dependent manner and blocked by protease inhibitors. We concluded that mbGluc was subjected to proteolytic cleavage with its transmembrane part staying in EV membrane and with its ectodomain being shed.

Shedding of membrane proteins is recognized as an important biological phenomenon playing role in development and in cancer progression^22,23^. The cleavage of the ectodomain can be catalyzed by various enzymes, including matrix metalloproteinases (MMPs) and a family of disintegrin and metalloproteinases (ADAMs). There are both secreted and membrane-anchored MMPs^24^. MMPs produced as pro-peptides are activated either intracellularly or in the extracellular space, with plasmin shown to be one of MMP activators^24,25^. MMPs, such as collagenases, digest the extracellular matrix and some proteins from this family are active against membrane proteins, such as growth factor receptors^24,26^. Another protein family, ADAMs, has also been described as potent inducers of ectodomain shedding. ADAM17 is responsible for cleavage of TNF-alpha and EGFR ligands^27,28^. MMPs and ADAMs have been identified in EVs in many studies both in cancer and normal cells^29–31^.

Proteins on the surface of EVs can also be subjected to ectodomain shedding as a result of proteolytic release. It has been postulated that this process can initiate even at the stage of EV formation in the endosome/Golgi apparatus^31^. The process was stimulated by the calcium ion influx triggered by ionomycin or other cleavage inducers. Proteins L1 and CD44 have been detected on the surface of EVs both in full-length and cleaved forms^31^. Correspondingly to our results, the cleavage of those proteins after EV release was time-dependent and reduced by protease inhibitors. Apoptosis and hypoxia were shown to be biological factors that promote ectodomain shedding^32^. The shedding of ectodomain of L1 and CD44 was mediated in part by ADAM10^31–33^. The phenomenon of L1 cleavage was also confirmed in EVs from the ascites fluid of patients affected with ovarian cancer^32^. Consistent with our results (Fig. 6), EpCAM was cleaved off EVs extracted from ascites fluid after exposure to serum in a time-dependent manner^34^. This suggests that serum-derived proteases are involved in the process of ectodomain shedding. We observed, however, that mbGluc also underwent cleavage after 24 hours in medium without FBS. This implies that enzymes on the membranes of EVs may be also involved. We detected a reduction of mbGluc cleavage by TAPI-0 (Fig. 6), which is an inhibitor of MMPs and membrane-imbedded ADAM17. The cleavage of EpCAM was also shown to be effectively inhibited by TAPI-0^34^. The broad-spectrum metalloproteinase inhibitor, galardin suppressed mbGluc shedding as well. It is likely that L1, CD44, EpCAM and the reporter protein mbGluc are examples of a large group of membrane proteins affected by ectodomain shedding. It is worth underscoring that fractionation of conditioned medium into EVs and non-vesicular components, including free protein complexes demonstrated an overlap for the majority of proteins^16^. This suggests that a lot of membrane proteins identified in EVs are subjected to shedding and hence are detectable in non-vesicular fractions. Taken together, the time-dependent and serum-enhanced shedding of mbGluc is probably mediated by different proteases, similarly to endogenous proteins previously described^31,32,34^.

The analysis of samples from an animal model revealed that only a small fraction (2–5%) of Gluc signal is attributable to cancer EVs in serum of tumor-bearing animals (Fig. 6J). In our previous study, we showed that mbGluc bioluminescence specific to EVs can be analyzed in animal serum by immunocapture with the use of antibodies targeting endogenous membrane antigens^8^. Our observation that around 95–98% of the membrane protein is detectable in a cleaved-off form is consistent with another study that demonstrated that EVs isolated from serum of breast cancer patients lost expression of EpCAM almost completely^34^. It is also possible that the method of EV isolation contributes to protein cleavage. This question seems worth exploring in future studies. Our observation is also in agreement with single EV analysis, which revealed that EVs originating from the same cell line rarely share the same antigen profile^35^. That high level of antigenic heterogeneity among EVs could also result from ectodomain shedding. Our results, along with previous reports, may have implications on design of diagnostic assays. Many EV isolation strategies applied in diagnostics are based on immunocapture^36^. Some assays may actually work by capturing the protein of interest, no matter whether it is associated with EVs or not. If exposed membrane protein antigens are shed from EVs it would make those approaches less efficient. Moreover, the level of proteolysis can vary from sample to sample which may result in increased assay variability or inability to capture EVs in some patient samples. An actual repertoire of membrane antigens on the surface of EVs depends both on the initial expression of proteins in the cell of origin, and on the proteolytic activity of plasma or other biofluids.

The properties of mbGluc described in this study make this reporter protein a good candidate for studying ectodomain shedding. So far, this phenomenon has been mainly investigated by analyzing protein sizes as shown by western blots. We propose the Triton X-114 separation combined with measurement of Gluc bioluminescence as a quantitative assay estimating proportions of a full-length membrane imbedded protein and soluble cleaved fragments. We have demonstrated that this strategy enables an evaluation of the impact of protease inhibitors on ectodomain shedding in EVs. Furthermore, we have shown that this assay can assess shedding of membrane proteins *in vivo*. mbGluc could be used as a measure of protein shedding under different conditions. Future research could look into how the tumor microenvironment affects loss of membrane antigens. For example, it has been shown that surface proteolysis of collagen XVII promotes invasiveness of squamous carcinoma cells^37^. Ectodomain shedding could be also a mechanism to loose cancer specific antigens and a route to evade immune system recognition and make immunotherapies less efficient^38^. Measuring proteolysis and how it can be targeted by therapeutics could be in the future facilitated by mbGluc assay. This method can also help in studying efficacy of various inhibitors of ectodomain shedding and how different transmembrane constituents influence susceptibility to protein cleavage. All those questions could be addressed thanks to the quantitative nature of the mbGluc assay and its functionality in *in vivo* models.

## Methods

### Cell culture

Human ovarian cancer cell line OVCAR5 (female) was obtained from American Type Culture Collection. A2780 (female) was acquired from European Collection of Cell Cultures. OVCA433 (female) ovarian serous adenocarcinoma cell line was a generous gift from Dr. Marcin Iwanicki (Stevens Institute of Technology, Department of Chemistry and Chemical Biology, NJ USA). OVCAR5 and A2780 were grown in RPMI-1640 medium (Corning, catalogue No. 10-040-CM). OVCA433 (Iwanicki *et al*., 2016)^39^ were cultured in 1:1 mixture of MCDB 105 (Cell Applications, catalogue No. 117–500) medium and Medium 199 (Gibco, catalogue No. 11150059). All media were supplemented with fetal bovine serum (FBS; Gemini Bio-products, catalogue No. 900–208) and penicillin-streptomycin solution (Corning, catalogue No. 30-002-Cl) at the final concentrations of 10% and 1%, respectively, unless stated otherwise. Cells were kept in a humidified incubator with 5% CO_2_ at 37 °C. All cells were tested for mycoplasma infection (MycoAlert Mycoplasma Detection Kit, Lonza, catalogue No. LT07-218) and found to be negative. Cells were counted by means of Bright-Line^™^ Hemacytometer (Sigma, catalogue No. Z359629).

### Animal protocol and collection of animal samples

Female athymic nude mice aged 5–7 weeks of weight 25–30 g were housed in the MGH Animal Facility and handled under the policies of the MGH Review Board. Animal protocols were approved by the Institutional Animal Care and Use Committee (IACUC) for the Massachusetts General Hospital (MGH) following the guidelines of the National Institutes of Health for the Care and Use of Laboratory Animals. Xenograft models were established by intraperitoneal injection of 3 × 10^6^ cells thoroughly washed free of the culture medium with cold PBS by three centrifugation cycles at 1,000 rpm for 5 min and resuspended in 1 mL PBS. Blood was collected by cardiac puncture at the time of animal sacrifice into BD Microtainer tube with no additive (Becton Dickinson, catalogue No. 365957). Blood was centrifuged at 1,200 × *g* for 15 min at room temperature. The supernatant was centrifuged at 1,200 × *g* for 5 min at room temperature and the supernatant of that spin was referred to as serum. Serum was stored in −80 °C and analyzed collectively after completion of the study.

### EV isolation from conditioned media

For the purpose of EV isolation, fetal bovine serum (Gemini Bio-products, catalogue No. 900–208) was EV-depleted by UC at 40,400 rpm (that corresponds to average RCF 120,101 × *g*) in 70 Ti rotor (fixed angle, average radius: 65.7 mm, k-Factor at maximum speed: 44, Beckman, catalogue No. 337922) in Optima L-90K ultracentrifuge (Beckman Coulter, catalogue No. 365670) for 17 hours at 4 °C and filtered through 0.22 μm (Millipore® catalogue No. SLGP033RS). To collect EVs, 10^6^ cells per flask were seeded in four to eight 75 cm^2^ flasks (Falcon® catalogue No. 353136) in 20 mL culture medium supplemented with FBS at 10% concentration. The medium was replaced after 24 hours with fresh medium with 5% EV-depleted FBS and collected 48 hours later when cells were at approximately 90% confluency. Cells were kept in a humidified incubator with 5% CO_2_ at 37 °C. This conditioned medium was subject to serial centrifugation steps combined with filtration: (1) 300 × *g* for 10 min at 4 °C (Thermo Scientific™ Sorvall™ Four-Place Swinging Bucket Rotor); (2) 2,000 × *g* for 10 min at 4 °C (Thermo Scientific™ Sorvall™ Four-Place Swinging Bucket Rotor); (3) filtration through 0.8 μm filter (Millipore®, catalogue No. SLAA033SS); (4) filtrate centrifuged at 40,400 rpm (that corresponds to average RCF 120,101 × *g*) in 70 Ti rotor for 2 hour at 4 °C with brake set to maximum (“0”) in polypropylene tubes (Beckman Coulter, catalogue No. 342414), (5) the pellet was resuspended in cold PBS previously filtered twice through 0.22 μm (Millipore® catalogue No. SLGP033RS) and centrifuged again at 40,400 rpm (that corresponds to average RCF 120,101 × *g*) in 70 Ti rotor for 2 hour at 4 °C in polypropylene tubes (Beckman Coulter, catalogue No. 342414), unless otherwise specified. The resulting suspension was referred to as the “120 K pellet”. This study followed MISEV criteria^40^ for nomenclature, collection from tissue culture conditioned media and EV isolation methods. We have submitted all relevant data of our experiments to the EV-TRACK knowledgebase (EV-TRACK ID: EV190080)^41^.

### Density gradient fractionation – sucrose and iodixanol

For top-loaded samples, the sucrose density gradient was formed by applying 875 μL sucrose solutions in the following order: 60% (bottommost), 45%, 30%, 8% (topmost) in Beckman polyallomer tube (Beckman, cat No. 326819). 500 μL sample (conditioned medium, EVs resuspended in double-filtered through 0.22 μm filter PBS) was loaded on top of the gradient. For bottom-loaded samples, the sample was mixed with 60% sucrose solution and applied at the bottom of the tube, with 45%, 30%, 8% (topmost) solutions that followed. The tubes were centrifuged in Optima MAX –XP centrifuge, in MLS-50 swinging bucket rotor (average radius: 71.7 mm, k-Factor at maximum speed: 71, Beckman, catalogue No. 367280) for 38 minutes or 18 hours (specified for each experiment) at 50,000 rpm (that corresponds to average RCF 200,620 × *g*) with brake set to 8 (very slow) at 4 °C. The fractions were collected from the top: 1) 500 μL (referred to as “Top” fraction); 2) ten fractions (350 μL) numbered 1-10. The density of fractions was determined after collection. For iodixanol gradient, OptiPrepTM Density Gradient Medium (Sigma, catalogue No. D1556) was dissolved in sterile water to concentrations 60%, 40%, 30%, 10% and stored in 4 °C. A sample (500 μL medium or EVs) was mixed with 875 μL 60% iodixanol, and loaded bottommost, which was followed by application of 875 μL iodixanol solutions: 40%, 30%, 10% (topmost) to reach a total volume of 4 mL.

### Size-exclusion chromatography (qEV column)

Cells were cultured in RPMI medium supplemented with 10% FBS for 24 hours. The medium was exchanged for RPMI supplemented with 5% EV-depleted FBS for next 48 hours. The conditioned medium was then centrifuged at 300 × *g* for 10 min at room temperature to remove floating cells. In experiments with down-stream western blot analysis, 10 mL supernatant was in addition concentrated on the filter (catalogue No. UFC910008, Millipore®) by centrifugation at 4000 × *g* for 20 minutes in a fixed angle rotor at 4 °C. 0,5 mL supernatant (or concentrate in case of filtered samples) was applied on the SEC column (qEVoriginal/70 nm, Izon) following the column equilibration procedure according to the manufacturer’s protocol. The sample was eluted by addition of PBS solution (freshly double filtered through 0.22 μm Millipore® filter, catalogue No. SLGP033RS). Thirty fractions of 500 μL were collected and subjected to bioluminescence assay or TCA protein precipitation (300 μL) for subsequent western blot analysis.

### Fast protein liquid chromatography

SEC was also performed on a high-resolution Akta Pure FPLC system (GE Health) with sequential Superdex 200 Increase 10/300 GL resin columns in triplicate. Approximately 1 mL of sample was injected into the FPLC system and 1.5 mL fractions were collected immediately. Total protein (BCA Assay, Pierce), total cholesterol (Raichem), phospholipids (phosphotidylcholine, Wako), and triglycerides (Pointe Scientific) were quantified using colorimetric assays. High-molecular weight standards were purchased from GE Healthcare.

### Triton X-114 phase separation

The procedure is based on the method described by Bordier (1981). A sample (volume 200 μL) was cooled down on ice for 30 minutes. Two μL cold Triton X – 114 (TX-114, Sigma, catalogue No. X114-100ML) was added to reach 1% concentration. A cushion (300 μL) of 6% sucrose/0.06% TX-114 in 10 mM Tris-HCl, pH 7.4, 150 mM NaCl was prepared in 1.7 ml Eppendorf. The sample with 1% TX-114 was overlaid on top of the cushion and incubated at 37 °C for 5 min. Aqueous (fraction B) and detergent phases (fraction A) were separated by centrifugation for 3 minutes at 300 × *g* at room temperature in a swinging bucket rotor. After centrifugation the detergent phase was found as an oily droplet at the bottom of the tube (fraction A). To achieve better isolation of phases, the aqueous phase (fraction B) was processed again (second cycle of separation). Fraction B was cooled-down on ice for 30 minutes, cold TX-114 was added to reach 0.5% solution (not counting previously contained TX-114) and was overlaid on a cushion of 6% sucrose/0.06% TX-114 in 10 mM Tris-HCl, pH 7.4, 150 mM NaCl. Following an incubation at 37 °C for 5 minutes, aqueous (fraction B1) and detergent (fraction B2) phases were separated by centrifugation for 3 minutes at 300 × *g* at room temperature in a swinging bucket rotor. The solution of 10 mM Tris-HCl, pH 7.4, 150 mM NaCl and TX-114 were added to all fractions (A, B1, B2) so that the volume and TX-114 concentration was the same. Bioluminescence assay to measure Gluc activity was performed in all A, B1 and B2 fractions in 5 technical replicates. “Fraction of water-soluble mbGluc [%]” (Fig. 6) was calculated as a ratio of bioluminescence in B1 to the sum of bioluminescence in A, B1 and B2 and expressed as a percentage. Proteins were precipitated by TCA method (decribed below) for subsequent western blot analysis from fractions A and B1 (referred to as “Det” and “Aq”, respectively in Fig. 6). The efficiency of isolation of aqueous and detergent phases increased with the number of separation cycles (Fig. S4). Two separation cycles were sufficient to investigate biological phenomena described in Fig. 6.

### Trichloroacetic acid (TCA) precipitation of proteins

A sample (300 μL), 100% ice cold acetone and 100% trichloroacetic acid (TCA) in liquid form were mixed in the 1:8:1 ratio. Following the precipitation for 1 hour at −20 °C, the samples were centrifuged at 18,000 × *g* for 15 minutes at 4 °C. The supernatant was discarded. The pellet was washed with 1 mL ice-cold acetone and centrifuged at 11,400 × *g* for 15 minutes at 4 °C. That washing step was repeated twice to remove TCA. Following the removal of acetone, the pellet was dried at room temperature and resuspended in lysis buffer - M-PER™ Mammalian Protein Extraction Reagent (Thermo Scientific, catalogue No. 78501) containing protease inhibitors (complete, Mini, Roche Diagnostics, catalogue No. 04693159001). The lysed samples were stored in the −80 °C until analysis by western blot.

### Transmission electron microscopy and immunolabeling

The 120 K pellet was centrifuged at 20,000 × *g* for 30 min at 4 °C and, after gentle removal of supernatant, fixed for 2 hours in 500 μL of 4% paraformaldehyde (32% aqueous solution, EM grade, Electron Microscopy Sciences, 15714-S) diluted in PBS. Fixed pellets were cryosectioned. Material from the SEC fractions was directly applied on the grid. Samples were immunolabeled with anti-Gluc (mouse; Nanolight) and anti-CD63 (mouse; BD Biosciences) followed by rabbit anti-mouse (Cappel/MP Biomedicals, LLC) and 5 nm protein A-gold (University Medical Center, Utrecht, The Netherlands). Images were captured using Tecnai G2 Spirit Bio TWIN transmission electron microscope.

### Bioluminescence assay

Gluc activity was measured in 10 μL samples in 3–5 technical replicates loaded onto a white 96-well luminometer plate (Greiner Bio-One International, catalogue No. 655075). After 17 s from automated injection at the rate 47 μL/s 50 μL of coelenterazine (Nanolight, 303–10, stock solution dissolved in methanol at 5 mg/mL) dissolved in PBS at 1.6 μg/mL, bioluminescence was detected during 1 s integration time in FlexStation 3 Reader (Molecular Devices). Alternatively, measurement of bioluminescence was performed after automated injection of coelenterazine at the rate 250 μL/s at 50 μg/mL in Synergy HTX multi-mode reader (Biotek Instruments).

### Transduction

After 24 hours of culture, the medium was replaced with the fresh culture medium with hexadimethrine bromide (Polybrene, Sigma, catalogue No. H9268) at a final concentration 4 μg/mL to enhance transduction efficiency. Previously described, mbGluc (CSCW-GlucB-IRES-GFP; Niers *et al*., 2012), Gluc, or palmGFP^14^ lentivirus vectors generated by MGH Vector Core (Boston, MA USA) were added to the media. mbGluc virus was added at MOI of 100, 10 and 1 in the experiment comparing the impact of transduction efficiency on the mode of mbGluc release. To promote virus infection, cells were centrifuged at 1,800 rpm (Thermo Scientific™ Sorvall™ Four-Place Swinging Bucket Rotor, radius 117 mm) for 90 min at 4 °C and transferred to the 37 °C incubator. Medium was exchanged the next day. Transduction efficiency was monitored by GFP signal using an inverted epifluorescence microscope (TE 200-U, Nikon, Melville, NY USA). After a few days of culture, depending on proliferation rate, cells were FACS sorted with regard to GFP expression to select population with a homogenous level of strong transgene expression.

### Preparation of beads covered with antibodies

Forty μl of beads in solution (Spherotech, Streptavidin polystyrene, SVP-50-5) per sample were washed four times with 1 mL 2% BSA (centrifugation at 3,000 × *g* for 5 min). 1.5 μg biotinylated antibodies were added to beads resuspended in 100 μL 2% BSA. Following antibodies were used in the assay: IgG1 (Biolegend, catalogue No. 400102, clone MOPC-21, unconjugated), anti-CD63 (Ancell, catalogue No. 215-030, clone AHN16.1/46-4-5, biotinylated), anti-bovine Apolipoprotein A-I rabbit polyclonal (MyBioSource, catalogue No. MBS1490491, unconjugated), anti-Gluc (rabbit polyclonal, Nanolight, catalogue No. 401 P, unconjugated). Unconjugated antibodies were biotinylated according to the procedure described below. The biotinylated antibodies were mixed with the beads overnight in HulaMixer® Sample Mixer (Thermo Fisher Scientific) at 4 °C. Beads with antibodies were washed four times with 1 mL 2% BSA (centrifugation at 3,000 × *g* for 5 min) leaving 100 μL solution after each washing step. Three hundred μL conditioned cell medium after cell removal (300 × *g* for 10 min at room temperature) were mixed with beads (in 100 μL 2% BSA) covered with antibodies overnight at 4 °C (cold room) in HulaMixer® Sample Mixer. After incubation with a sample, beads were washed four times with 1 mL 2% BSA (centrifugation at 3,000 × *g* for 5 min). The beads were then resuspended in PBS to reach the same volume in all tubes. Volumes of 10 μL were used for bioluminescence assay in 3–5 technical replicates.

### Biotinylation of antibodies

The antibodies were biotinylated by *EZ-Link* Sulfo-NHS-biotin (10 mM, Pierce, catalogue No. 21217) solution in PBS, following manufacturer’s instructions. Briefly, the mixture of antibody and EZ-Link Sulfo-NHS-biotin was incubated overnight at 4 °C. Unreacted Sulfo-NHS-biotin was removed using a Zeba spin desalting column (7 K MWCO, Thermo Scientific, catalogue No. 89882).

### Western blot

Samples were lysed in M-PER™ Mammalian Protein Extraction Reagent (Thermo Scientific, catalogue No. 78501) buffer containing protease inhibitors (complete, Mini, Roche Diagnostics, catalogue No. 04693159001). Protein concentration was determined by BCA protein assay kit (Pierce, catalogue No. 23225). Thirty microliters of each sucrose fraction were incubated with 30 μL M-PER™ Mammalian Protein Extraction Reagent. Twenty microliters of that solution were boiled for 5 min in SDS sample buffer (Boston BioProducts, catalogue No. BP-110R). Samples precipitated by TCA method were normalized to input total protein. Thirty micrograms of total protein were boiled for 5 min in SDS sample buffer. Proteins were resolved by the NuPAGE^TM^ gradient 4%-12% Bis-Tris Gel (Invitrogen, catalogue No. NP0321BOX) with molecular weight standards (Precision Plus Protein Standards, Bio-Rad, catalogue No. 161–0374), and transferred onto nitrocellulose membranes (0.2 μm, Bio-Rad, catalogue No. 162–0112). The membranes were blocked with 5% non-fat milk (LabScientific, catalogue No. M08425) and incubated overnight with anti-Gluc (rabbit polyclonal, Nanolight, catalogue No. 401 P, unconjugated) antibody at the dilution 1:1000, anti-ApoA-I (rabbit anti-bovine polyclonal, MyBioSource, catalogue No. MBS1490491, unconjugated) antibody at the dilution 1:500, anti-GFP (rabbit polyclonal, Thermo Fisher Scientific, catalogue No. A11122, unconjugated) antibody at the dilution 1:1000. This was followed by binding of secondary antibodies conjugated to horseradish peroxidase (HRP; donkey, anti-Rabbit IgG, GE Healthcare, NA934-1ML) at the dilution 1:7500 and signal detection with a chemiluminescent substrate (SuperSignal West Pico Chemiluminescent Substrate, Thermo Scientific, catalogue No. 34077).

### Statistical analysis

Analysis of data was performed in R programming language (version 3.4.1) using RStudio (version 0.98.1060). Normality of distribution was verified with Shapiro test. Groups with normal distribution were compared with t-Student test. Groups with distribution deviating from normal were compared using Mann-Whitney test. A paired test (Wilcoxon signed rank test or t-Student) was performed to compare data from biological replicates. Plots were generated using ggplot2 and ggpubr R packages. The results were considered significant for *p* values < 0.05. *P* values were either specified in the figure or denoted as asterisks: ns – p > 0.05, *p < 0.05; **p < 0.01; ***p < 0.001, ****p < 0.0001. Plot whiskers extend to the most extreme data point which is within 1.5 times the interquartile range from the box in the boxplots. Plot whiskers extend to standard deviation from the bar in the barplots. Numbers of replicates are stated in the figure legends.

## Supplementary information

Supplementary Figures S1-S5

## Data Availability

All data generated or analyzed during this study are included in this published article (and its Supplementary Information files).
