# Supplementary material for: Membrane-bound Gaussia luciferase as a tool to track shedding of membrane proteins from the surface of extracellular vesicles
Source: Sci Rep. 2019 Nov 22;9:17387. doi: 10.1038/s41598-019-53554-y (PMC6874653; doi:10.1038/s41598-019-53554-y)
Supplement: Supplementary file 1 — Supplementary Figures S1-S5 [file 41598_2019_53554_MOESM1_ESM.pdf]

Membrane-bound Gaussia luciferase as a tool to track shedding of membrane proteins from the surface of extracellular vesicles

Mikołaj Piotr Zaborowski[1,2,3,*], Pike See Cheah[1,2,4], Xuan Zhang[1,2], Isabella Bushko[1,2], Kyungheon Lee[5], Alessandro Sammarco[1,2,6], Valentina Zappulli[1,2,6], Sybren Lein Nikola Maas[1,2,7], Ryan M. Allen[8], Purva Rumde[1,2], Bence György[1,2,9], Massimo Aufiero[1,2], Markus W. Schweiger[1,2], Charles Pin-Kuang Lai[1,2,10], Ralph Weissleder[5,11,12], Hakho Lee[5,11], Kasey C. Vickers[8], Bakhos A. Tannous[1,2,11], Xandra O. Breakefield[1,2,11,*]

[1]Department of Neurology, Massachusetts General Hospital, Charlestown, MA 02129 USA
[2]Program in Neuroscience, Harvard Medical School, Boston, MA 02115 USA
[3]Department of Gynecology, Obstetrics and Gynecologic Oncology, Division of Gynecologic Oncology, Poznan University of Medical Sciences, 60-535 Poznań, Poland
[4]Department of Human Anatomy, Faculty of Medicine and Health Sciences, Universiti Putra Malaysia, 43400, Serdang, Selangor, Malaysia
[5]Center for Systems Biology, Massachusetts General Hospital, Boston, MA 02114 USA
[6]Department of Comparative Biomedicine and Food Science, University of Padua, Padua, Italy
[7]Department of Neurosurgery, UMC Utrecht Brain Center, University Medical Center, Utrecht University, 3584 CX, Utrecht, The Netherlands
[8]Department of Medicine, Vanderbilt University Medical Center, Nashville, TN 37232 USA
[9]Institute of Molecular and Clinical Ophthalmology Basel, 4031 Basel, Switzerland
[10]Present address: Institute of Atomic and Molecular Sciences, Academia Sinica, Taipei 10617, Taiwan
[11]Department of Radiology, Massachusetts General Hospital, Boston, MA 02114 USA
[12]Department of Systems Biology, Harvard Medical School, Boston, MA 02115 USA
[*]Corresponding authors: breakefield@hms.harvard.edu, mikolaj.zaborowski@gmail.com (ORCID: 0000-0002-4400-6688)

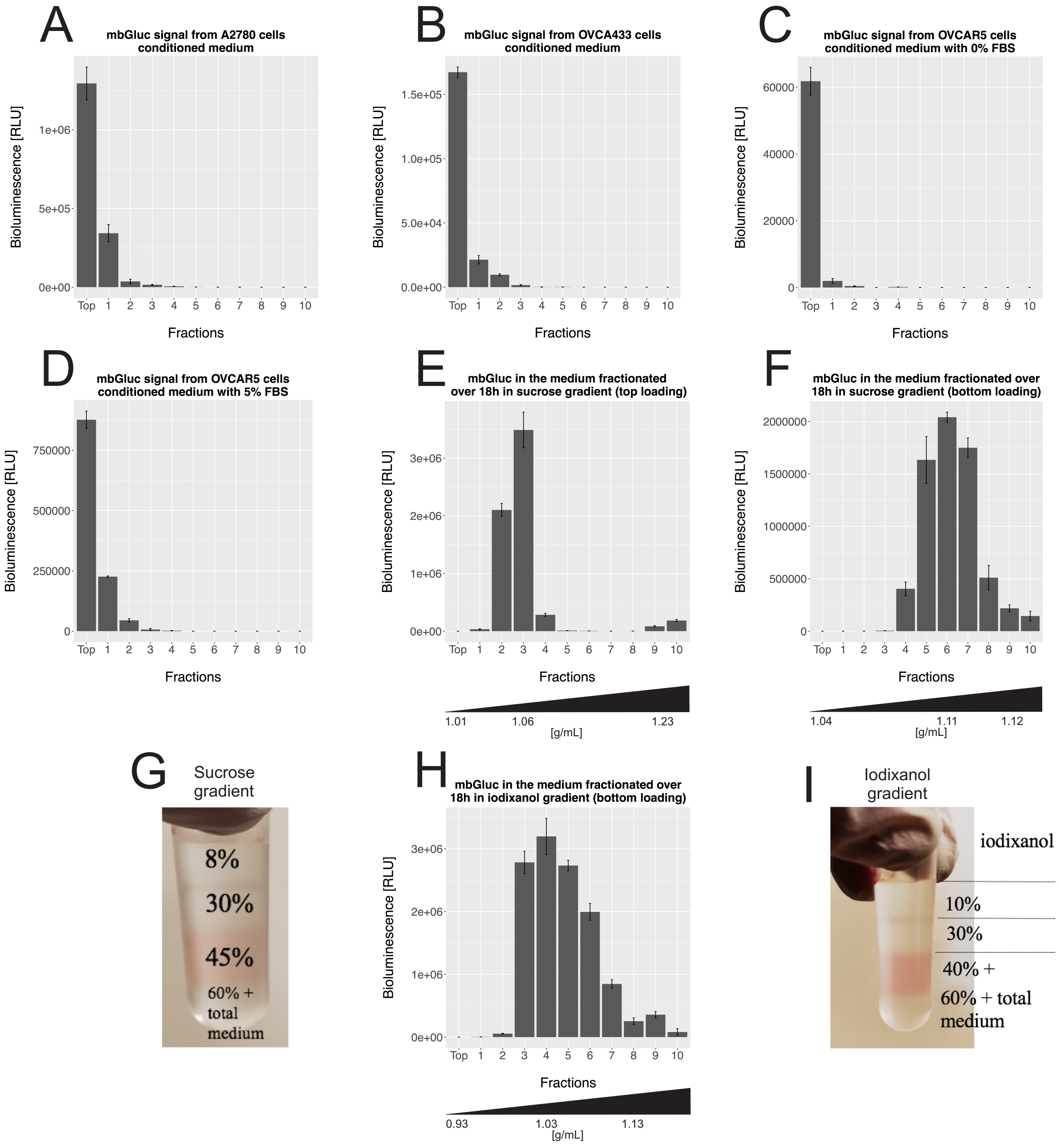
A
mbGluc signal from A2780 cells conditioned medium
Bioluminescence [RLU]
Fractions
B
mbGluc signal from OVCA433 cells conditioned medium
Bioluminescence [RLU]
Fractions
C
mbGluc signal from OVCAR5 cells conditioned medium with 0% FBS
Bioluminescence [RLU]
Fractions
D
mbGluc signal from OVCAR5 cells conditioned medium with 5% FBS
Bioluminescence [RLU]
Fractions
E
mbGluc in the medium fractionated over 18h in sucrose gradient (top loading)
Bioluminescence [RLU]
Fractions
1.01
1.06
1.23
[g/mL]
F
mbGluc in the medium fractionated over 18h in sucrose gradient (bottom loading)
Bioluminescence [RLU]
Fractions
1.04
1.11
1.12
[g/mL]
G
Sucrose gradient
8%
30%
45%
60% + total medium
H
mbGluc in the medium fractionated over 18h in iodixanol gradient (bottom loading)
Bioluminescence [RLU]
Fractions
0.93
1.03
1.13
[g/mL]
I
Iodixanol gradient
iodixanol
10%
30%
40% +
60% + total medium

Figure S1.

Figure S1. mbGluc Is Abundant in Low Density Fractions of Step-Gradient. Related to Figure 2. (**A**) mbGluc activity after fractionation of A2780 cells conditioned medium in sucrose density gradient (procedure in Fig. 2A, sample loaded on top of the gradient, 38 minutes spin at 200K × *g*, culture medium supplemented with 5% EV-depleted FBS). (**B**) mbGluc activity after fractionation of OVCA433 cells conditioned medium in sucrose density gradient (procedure in Fig. 2A, sample loaded on top of the gradient, 38 minutes spin at 200K × *g*, culture medium supplemented with 5% EV-depleted FBS). (**C**) mbGluc activity after fractionation of OVCAR5 cells conditioned medium in sucrose density gradient (procedure in Fig. 2A, sample loaded on top of the gradient, 38 minutes spin at 200K × *g*, culture medium supplemented with 0% EV-depleted FBS). (**D**) mbGluc activity after fractionation of OVCAR5 cells conditioned medium in sucrose density gradient (procedure in Fig. 2A, sample loaded on top of the gradient, 38 minutes spin at 200K × *g*, culture medium supplemented with 5% EV-depleted FBS). (**E**) mbGluc activity after fractionation of OVCAR5 cells conditioned medium in sucrose density gradient (sample loaded on top of the gradient, centrifugation for 18 hours at 200K × *g*, culture medium supplemented with 5% EV-depleted FBS). (**F**) mbGluc activity after fractionation of OVCAR5 cells conditioned medium in sucrose density gradient (sample loaded at the bottom of the gradient, centrifugation for 18 hours at 200K × *g*, culture medium supplemented with 5% EV-depleted FBS). (**G**) OVCAR5 conditioned medium loaded at the bottom of the sucrose density gradient. Medium was mixed with 60% sucrose solution. Loading of 45% solution on that mixture resulted in the move of medium dye to the top of 45%+60% solution (picture before centrifugation). (**H**) mbGluc activity after fractionation of OVCAR5 cells conditioned medium in iodixanol density gradient (sample loaded at the bottom of the gradient, centrifugation for 18 hours at 200K × *g*, culture medium supplemented with 5% EV-depleted FBS). (**I**) OVCAR5 conditioned medium loaded at the bottom of the iodixanol density gradient. Medium was mixed with 60% iodixanol solution. Loading of 40% solution on that mixture resulted in the move of the pink medium dye to the top of 40%+60% mixed solution (picture before centrifugation).

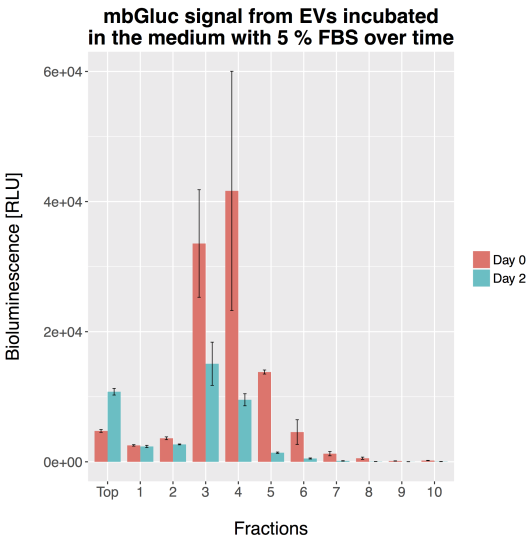

Figure S2. mbGluc as a Measure of Release of EV-bound Membrane Proteins. Related to Figure 3. mbGluc activity measured after fractionation of EVs derived from OVCAR5 mbGluc-positive cells in sucrose density gradient following incubation at 37°C in medium supplemented with 5% EV-depleted medium for 0 and 2 days.

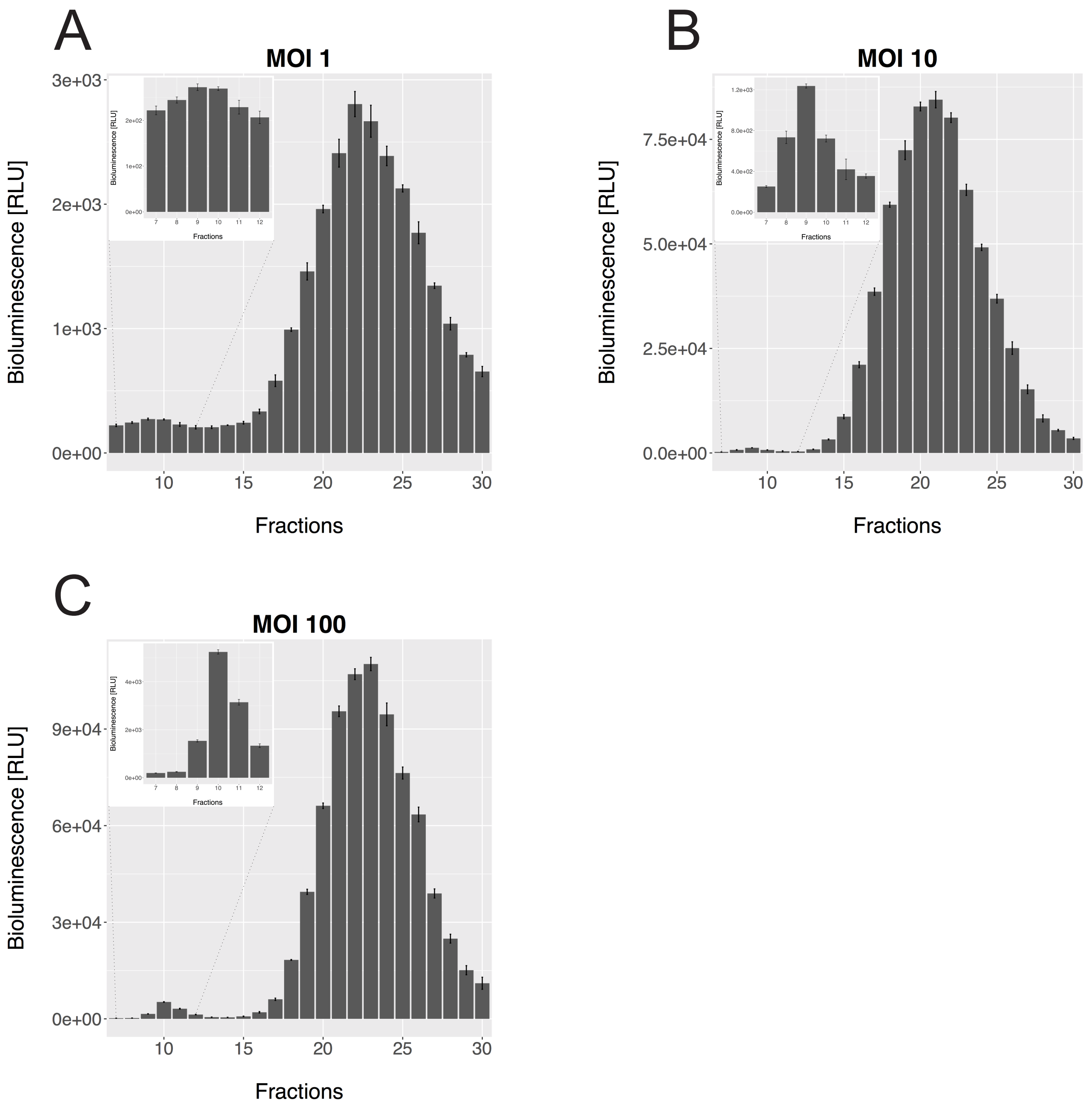

Figure S3. Level of Expression Does Not Affect Mode of Release of mbGluc. Related to Figure 4. (**A**) mbGluc activity in SEC fractions (qEV column) after fractionation of OVCAR5 medium following transduction with a lentivirus at MOI 1 (Procedure in Figure 4A). (**B**) mbGluc activity in SEC fractions of OVCAR5 medium following transduction with a lentivirus at MOI 10. (**C**) mbGluc activity in SEC fractions of OVCAR5 medium following transduction with a lentivirus at MOI 100. The insert represents fractions 7-12 with a modified scale on Y axis.

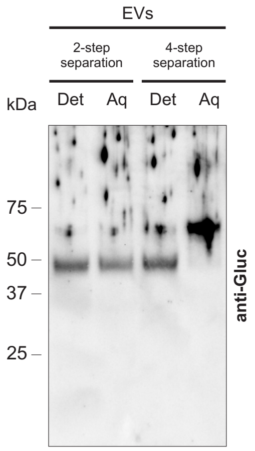

Figure S4. Increased number of centrifugation steps in TX-114 procedure improves separation of EV-bound mbGluc. Related to Figure 6.
Western blot of mbGluc protein isolated from OVCAR5-mbGluc+ EVs isolated by UC subjected to TX-114 procedure that included either two or four separation cycles.

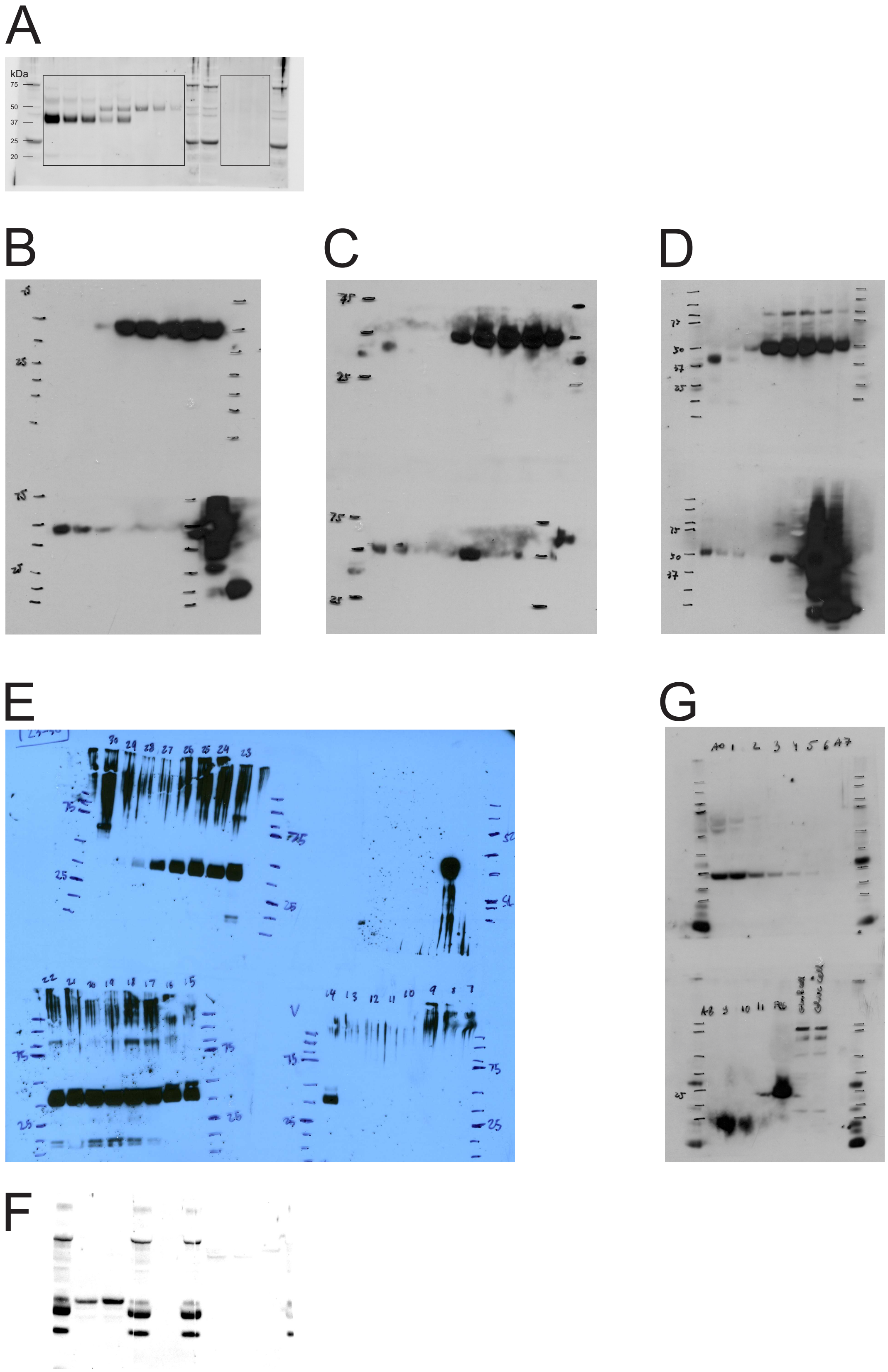

Figure S5. Full-length gels.
(**A**) Full picture of the gel presented in Fig. 2C. (**B**) Full picture of the gel presented in Fig. 3C (upper panel). (**C**) Full picture of the gel presented in Fig. 3C (middle panel). (**D**) Full picture of the gel presented in Fig. 3C (lower panel). (**E**) Full picture of the gel presented in Fig. 4C. (**F**) Full picture of the gel presented in Fig. 4G. (**G**) Full picture of the gel presented in Fig. 5C.
